# Salvage Therapy of Multiple Myeloma: The New Generation Drugs

**DOI:** 10.1155/2014/456037

**Published:** 2014-05-19

**Authors:** Alessandra Romano, Concetta Conticello, Maide Cavalli, Calogero Vetro, Cosimo Di Raimondo, Valentina Di Martina, Elena Schinocca, Alessia La Fauci, Nunziatina Laura Parrinello, Annalisa Chiarenza, Francesco Di Raimondo

**Affiliations:** ^1^Hematology Section, Department of Clinical and Molecular Biomedicine, University of Catania, Via Citelli 6, 95124 Catania, Italy; ^2^Fondazione Veronesi, Via Lancellotti 18, 00186 Roma, Italy; ^3^UOC di Ematologia con Trapianto di Midollo Osseo, Ospedale Ferrarotto, Via Citelli 6, 95124 Catania, Italy

## Abstract

During the past decade, overall results of treatment of multiple myeloma (MM) have been improved and survival curves are now significantly better with respect to those obtained with historical treatment. These improvements are linked to a deeper knowledge of the biology of disease and to the introduction in clinical practice of drugs with different mechanism of action such as proteasome inhibitors and immunomodulatory drugs (IMiDs). However, MM remains in most cases an incurable disease. For patients who relapse after treatment with novel agents, the prognosis is dismal and new drugs and therapeutic strategies are required for continued disease control. In this review, we summarize new insights in salvage therapy for relapsed/refractory MM as emerging from recent clinical trials exploring the activity of bendamustine, new generation proteasome inhibitors, novel IMiDs, monoclonal antibodies, and drugs interfering with growth pathways.

## 1. Introduction

During the past decade, overall results of treatment of multiple myeloma (MM) have been improved and survival curves are now significantly better with respect to those obtained with historical treatment. These improvements are linked to a deeper knowledge of the biology of disease and to the introduction in clinical practice of drugs with different mechanism of action such as proteasome inhibitors (bortezomib, carfilzomib) and immunomodulatory drugs (IMiDs; thalidomide, lenalidomide, and pomalidomide) [1].

However, MM remains in most cases an incurable disease, and new drugs and therapeutic strategies are required for continued disease control. In this perspective, several new drugs are currently undergoing evaluation, and many appear very promising on the basis of reported initial results [2, 3].

The natural history of MM includes recurrence of active disease defined as relapse when salvage treatment is needed after an off-therapy period, or refractory disease if nonresponsive while on salvage therapy, or progressing within 60 days of last therapy (see the following part, [4]).

*Definition of Progressive Disease in accord with MM Uniform Reporting of Clinical Trials, Report of the 2008 International Myeloma Workshop Consensus*:

*Definitions of Relapsed/Refractory MM*

 
*Refractory myeloma*. It is a disease that is nonresponsive while being on salvage therapy or progresses within 60 days of last therapy.
 
*Primary refractory myeloma*. It is a disease that is nonresponsive in patients who have never achieved a minor response with any therapy.
 
*Relapsed myeloma*. After a period of being off therapy, it requires the initiation of salvage therapy.
 
*Relapsed-and-refractory myeloma*. It is nonresponsive while being on salvage therapy (achieved minor response (MR) or better at some point in their disease course).

Most of the studies reporting results of the new drugs are difficult to interpreter because they are small single arm studies, they deal with very heterogeneous groups of patients, and quite often relapsed and refractory patients are lumped together. In addition, there is a lack of information about the natural history of MM in the relapsed setting, after exposure to novel agents.

A recent study described the poor outcome of patients who are refractory to current treatments and provided context for interpreting trials of new drugs [5]. This study included 286 patients with relapsed MM, who were refractory to bortezomib and were relapsed following an IMiD. Median age at diagnosis was 58 years, and time from diagnosis to salvage treatment (T0) was 3.3 years. The first regimen contained bortezomib in 26% patients and an IMiD in 33% of patients. A minor or better response was achieved after at least one therapy after T0 in 44% of patients, including partial response (PR) in 32%. The median overall survival (OS) and event-free survival (EFS) from T0 were 9 and 5 months, respectively.

Thanks to new information on the biology of MM plasma cells and deeper knowledge of the metabolic pathways that the neoplastic cells use for their growth, new drugs have been developed. However, the new therapeutic strategies for relapsed and refractory patients arise not only from drugs with a new mechanism of action, targeting the deregulated pathways in MM, but also from analogs of agents already approved for treatment of MM such as new chemotherapy drugs, second and third generation of proteasome inhibitors, and new IMiDs.

In addition, it is not clear if the novel drugs should be used in monotherapy or in combination. Looking at the emerging tumor biology, combination therapy has the potential benefit of suppressing and eliminating more subclones at the same time, but patients with refractory disease may be more debilitated and unable to tolerate aggressive combination treatments. Among patients with early relapse, the use of a 3-drug regimen (bortezomib, thalidomide, and dexamethasone) improved overall response rate (ORR), depth of response, and progression-free survival with a trend toward improved overall survival [6]. Ongoing phase 3 studies will provide further evidence to address if 3-drug based regimens are better than 2 drugs, using other new agents such as carfilzomib (carfilzomib/lenalidomide/dexamethasone versus lenalidomide/dexamethasone), panobinostat (panobinostat/bortezomib/dexamethasone versus bortezomib/dexamethasone), elotuzumab (elotuzumab/lenalidomide/dexamethasone versus lenalidomide/dexamethasone), and pomalidomide (pomalidomide/bortezomib/dexamethasone versus bortezomib/dexamethasone).

## 2. Novel Alkylators

Alkylators, such as melphalan and cyclophosphamide, are the backbone for the combination with the new drugs, although eventually patients develop resistance against them and the search for new alkylating agents is fully justified. In this scenario, MM researcher has shown an increased interest for bendamustine, an older alkylating drug developed behind the Iron Curtain but only recently used in Western countries.

Bendamustine is a nitrogen mustard with both purine analogue and alkylating cytotoxic effects for its unique chemical structure: a 2-chloroethylamine alkylating group (like melphalan and cyclophosphamide), a benzimidazole ring, and a butyric acid side chain (like in chlorambucil) [25]. Therefore, bendamustine can activate apoptosis and inhibit mitotic checkpoints instead of inducing necrosis alone, as all other alkylators do.

Bendamustine is effective as a single agent and in several combinations with novel agents (bortezomib, thalidomide, and lenalidomide) for the treatment of relapsed/refractory MM (Table 1).

In a phase I study involving 31 patients with progressive disease after autologous stem cell transplantation (ASCT), bendamustine was tested starting from 60 mg/m^2^ on days 1 and 2 of each 28-day cycle. Dose-limiting toxicity of febrile neutropenia developed in one patient after bendamustine 100 mg/m^2^ with an overall response rate (ORR) of 55%. Median duration of response was 8 months and median progression-free survival (PFS) for the whole study population was 26 weeks and for the patients receiving 90 or 100 mg/m^2^ it was 36 weeks [26].

A phase I-II study including 40 MM patients previously exposed to bortezomib or alkylators tested the association of escalating doses of bendamustine 50, 70, or 90 mg/m^2^ (days 1 and 4) plus bortezomib 1.0 mg/m^2^ (days 1, 4, 8, and 11) for up to eight 28-day cycles [7]. The bendamustine MTD was 90 mg/m^2^. The most common grade 3/4 adverse events were leucopenia (58%), neutropenia (50%), lymphopenia (45%), and thrombocytopenia (30%). ORR was 48% (one CR, two VGPR, nine PR, and seven MR) for all 40 enrolled patients, 52% (16/31) at the MTD [7].

After these encouraging results, phase II studies tested the efficacy of bendamustine in combination with bortezomib and steroids.

In 79 patients with relapsed/refractory MM, bendamustine was given at 70 mg/m^2^on days 1 and 4, bortezomib 1.3 mg/m^2^ on days 1, 4, 8, and 11, and dexamethasone 20 mg on days 1, 4, 8, and 11, every 28 days up to eight cycles (BBD). With this regimen, grades 3 and 4 anemia and leukopenia were seen in 18.7% of patients, while grade 4 thrombocytopenia was observed in 6%. Grades 3 and 4 infections were observed in 20% of patients, with two deaths (3%) because of infection. In addition, a doubling of the incidence of self-assessed grade 2 neurotoxicity from baseline to cycle 8 was documented and grade 3-4 neuropathy was observed in 7% of patients at the last treatment cycle. BBD induced a very fast response with an ORR of 75.9% (including 15% CR), and PFS was 9.7 months. Preexposure to lenalidomide was correlated with a lower response rate and shorter time to progression (TTP). Of interest, incidence of response, its duration, and OS were not different between patients defined as low risk or high risk according to cytogenetics [8].

Similar results were obtained from the Italian group evaluating bendamustine plus bortezomib and dexamethasone (BVD) every 28 days for the first 6 cycles and then every 56 days for 6 further cycles. Seventy-five patients with relapsed/refractory MM, treated with ≤4 prior therapies and not refractory to bortezomib, were treated. Grade 3-4 hematologic toxicities (thrombocytopenia and neutropenia), neuropathy (8%), and gastrointestinal and cardiovascular events were the more frequent side effects. The ORR was 71.5% (including 16% CR). TTP was 16.5 months at a median follow-up of 12 months and 1-year overall survival was 78% [9].

In a multicentric study involving 78 relapsed-refractory MM patients BVP consisted of bendamustine 60 mg/m^2^ on days 1 and 2, bortezomib 1.3 mg/m^2^ on days 1, 4, 8, and 11, and prednisone 100 mg on days 1, 2, 4, 8, and 11. ORR was 69% with PFS of 11 months, superior to not-heavily pretreated patients [27]. BVP was effective also in the cohort of 36 patients with light chain-induced renal failure (creatinine clearance <60 mL/min), achieving ORR 67% [28].

The optimal dose of bendamustine associated with thalidomide is 60 mg, as shown by two independent phase 1-2 studies [10, 11]. In phase 1 study bendamustine 60 mg plus prednisone 100 mg was tested in association with increasing doses of thalidomide (50, 100, and 200 mg), without achieving the thalidomide MTD in 28 relapsed/refractory patients. Twenty-four patients responded after at least two cycles (4 CR, 6 VGPR, and 14 PR), with ORR 80% and PFS of 11 months. Only mild/moderate nonhematological side effects were observed and no patient developed dose-limiting hematotoxicity [10].

Two recent phase 1-2 studies identified MTD of bendamustine as 75 mg/m^2^ when associated with lenalidomide. In the American study, involving 29 patients relapsed after a median number of 3 previous treatments, bendamustine 75 mg/m^2^ (days 1 and 2) plus lenalidomide 10 mg (days 1–21), and weekly dexamethasone 40 mg resulted in ORR 76% and PFS of 6.1 months. Hematological toxicity, including 3-4 grade neutropenia, thrombocytopenia, and anemia, was responsible for discontinuation of treatment in one-third of patients. Nonhematological side effects included fatigue, diarrhea, hypocalcemia, hyperglycemia, and nausea [12].

In the German study, enrolling 21 patients in five cohorts treated with bendamustine (60 up to 75 mg/m^2^), lenalidomide (10 up to 25 mg), and prednisone 100 mg, the MTD was not reached. Authors suggested the best schedule as bendamustine 75 mg/m^2^ on days 1-2, lenalidomide 25 mg on days 1–21 every 28 days, and prednisone 100 mg on days 1–4, to achieve ORR 76% and PFS of 48% at 18 months [29].

Thus, bendamustine is effective in monotherapy and in combination with novel agents at reduced doses to limit toxicity but longer follow-up is needed to overcome the lack of information on overall survival.

## 3. Novel Proteasome Inhibitors

Bortezomib is a dipeptide boronic analog that reversibly inhibits the chymotryptic activity of the 20S subunit of the proteasome [30]. Resistance to bortezomib has been documented both in vitro and in vivo, due to upregulation of the proteasome subunits, mainly for increased levels of the *β*5-subunit. However, there is no clear quantitative correlation between level of resistance and the extent of *β*5-subunit expression. Moreover, although specific variants of the proteasome genes which encode the *β* subunits of the 20S proteasome (PSMB5) have been previously identified in preclinical models of bortezomib resistance, these variants were not detected in patient tumor samples collected after clinical relapse from bortezomib, which suggests that alternative mechanisms may underlie bortezomib lack of sensitivity [31].

**Figure 1:**
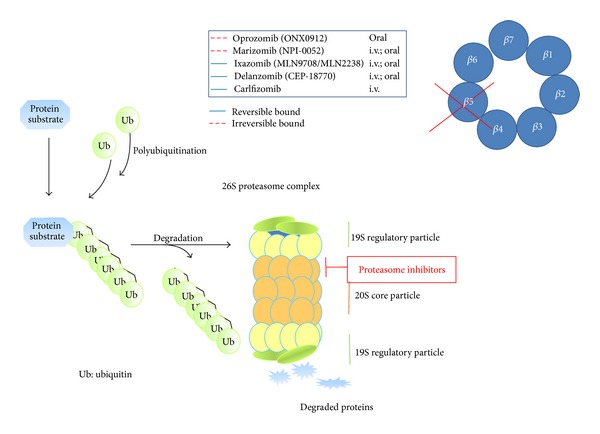


To overcome resistance to bortezomib, second and third generations of proteasome inhibitors have been developed, characterized by an irreversible bond to *β*5-subunit (such as carfilzomib) or binding to different subunits (such as marizomib) or the possibility of oral administration (such as ixazomib, Table 2 and Figure 1).

Carfilzomib gained FDA approval in 2012 for MM patients who have received at least two prior therapies, including bortezomib and an IMiD, and have demonstrated disease progression on or within 60 days of completion of the last therapy, as consequence of promising findings of four phase 2 clinical trials (PX-171-003-A0, PX-171-003-A1, PX-171-004, and PX-171-005).

In PX-171-003-A0, 20 mg/m^2^ carfilzomib was evaluated in 46 refractory patients, with a median of 3 cycles administered [32]. Infusion reactions were observed including fever, chills, myalgia, facial swelling or flushing, vomiting, weakness, hypotension, chest tightness, or shortness of breath. Thus, prophylactic premedication with very low-dose dexamethasone (4 mg) prior to carfilzomib in cycles 1 and 2 is recommended to decrease the incidence and severity of infusion reactions.

In PX-171-003-A1, carfilzomib was tested at 20 or 27 mg/m^2^ as single agent using dexamethasone as premedication in 257 patients with relapsed/refractory MM, who responded to at least one therapy. Median number of previous treatment was five, including IMiD and bortezomib and 73% of bortezomib-refractory patients [33]. ORR was 36%, with 5% VGPR (Table 3). Cytogenetic profile was available for 229 patients. ORR was comparable between patients with standard and high-risk profile, the subgroups (25.8% versus 24.6%), while time-to-event endpoints showed a trend of shorter duration in high-risk patients, including median duration of response (5.6 months versus 8.3 months) and overall survival (9.3 versus 19.0 months). Thus, carfilzomib seems to partially overcome the impact of high-risk cytogenetics on heavily pretreated patients [13].

In the subsequent study PX-171-004, 164 relapsed/refractory patients, previously treated with more than one but less than three therapies and bortezomib naïve, received carfilzomib as single agent (20 or 27 mg/m^2^) [34].

In PX-171-005, 50 relapsed/refractory MM patients with renal insufficiency received carfilzomib in monotherapy. The dose was safely escalated to the target dose (from 15 to 20 to 27 mg/m^2^) used in patients with normal renal function. Carfilzomib did not appear to be associated with clinically relevant nephrotoxicity, and most patients who experienced irreversible worsening of renal function had clear evidence of progressive MM [35].

Then, a cross-trial analysis examined the safety profile of single-agent carfilzomib in 526 patients with relapsed and/or refractory MM in the above-mentioned four phase II trials upon which US approval was based [36]. Overall, the most common adverse events of any grade were fatigue, anemia, and nausea. Aggregated cardiac-failure events (including congestive heart failure, pulmonary edema, and decreased ejection fraction) were reported in 7.2%, regardless of causality. The mortality rate was the same (7%) in patients who had baseline cardiac risk factors as it was for patients without these risk factors. Carfilzomib is not actually contraindicated in cardiopathic patients but anecdotic reports suggest a warning in elderly patients. Serial echocardiograms are being conducted in a number of ongoing carfilzomib studies, including a substudy in the randomized phase III trial ENDEAVOR (ClinicalTrials.gov identifier: NCT01568866), which is comparing carfilzomib and dexamethasone versus bortezomib and dexamethasone. Dyspnea, a common complication from the disease itself and from other MM treatments, was reported in 42.2% of patients; however, most incidences were grade 1 or 2 and transient and resolved without dose reduction or discontinuation. There are concerns that dyspnea may develop due to fluid overload, recommended up to 250 mL saline to prevent tumour-lysis syndrome rather than drug toxicity. Hydration has been previously recommended with carfilzomib treatment due to concerns of acute deterioration of renal function [36].

Higher doses of carfilzomib (up to 56 mg/m^2^) can be safely infused over 30 minutes and are currently under investigation (PX-171-007, presented in abstract form at ASH 2011). In the ongoing trial ENDEAVOR mentioned above, 56 mg/m^2^ carfilzomib is given in combination with dexamethasone in relapsed patients. As shown by three phase-2 studies, carfilzomib activity in monotherapy is inversely correlated with the numbers of previous treatment.

Then, carfilzomib has been tested in association with IMiDs, lenalidomide, and pomalidomide.

The combination of carfilzomib with lenalidomide and low dose dexamethasone (Rd) has shown promising results and appears to be well tolerated in 51 patients affected by relapsed MM after 1–3 prior treatments (75% previously treated with bortezomib) [14]. ORR was 78%, including 41% ≥ VGPR (Table 3). Adverse events were mainly hematological with grade 3-4 anemia, thrombocytopenia, and neutropenia. Some patients (7.5%) experienced grade 3 or 4 fatigue. Shortness of breath as well as hypertension and rare cases of significant cardiac dysfunction were reported. Due to this promising findings, the combination is being further explored in patients with relapsed MM in the randomized phase III trial ASPIRE (ClinicalTrials.gov identifier: NCT01080391), but no data are currently available.

The combination of carfilzomib with pomalidomide and low dose of dexamethasone (Car-Pom-d) was investigated in a multicenter phase I/II trial involving 82 heavily pretreated, lenalidomide-refractory MM patients [15]. The MTD was carfilzomib 27 mg/m^2^ on days 1-2, 8-9, and 15-16, pomalidomide 4 mg on days 1–21, and dexamethasone 40 mg on days 1, 8, 15, and 22. Seventy-nine patients with a median of five previous treatments were evaluable for Car-Pom-d clinical activity. The regimen was tolerated well with no unexpected toxicity. Toxicities were generally reversible and manageable with G3-4 neutropenia in one-third of patients and limited G3-4 nonhematological toxicities. Nonhematological adverse events were mainly grade fatigue and dyspnea. However, a case of fatal pneumonia and a case of fatal pulmonary embolism related to treatment occurred. The ORR was 70% (with 27% VGPR, Table 3) with a median duration of response of 17.7 months. Additional 13% of patients experienced a minimal response for a total clinical benefit rate of 83% and a PFS of 9.7 months. The combination was independent from FISH/cytogenetic risk status evaluated according to mSMART profile [37]. Dr. Shah (ASH 2013, abstract number 690) gave an update of the phase I/II dose expansion trial at last ASH annual conference.

Among the new generation of proteasome inhibitors with promising activity in relapsed/refractory, certainly Ixazomib (MLN9708) deserves a mention since it is an oral, reversible, specific 20S proteasome inhibitor, currently tested in clinical trials, with promising activity and a favorable profile of tolerability.

A phase I study evaluated single-agent ixazomib given to 32 patients on days 1, 8, and 15 of a 28-day cycle, for up to 12 cycles. After MTD was assessed at 2.97 mg/m^2^, ixazomib was tested in an expansion cohort of further 31 previously treated patients (median 4 lines). In this last cohort, ORR was 26%, including 25% PR (Table 3, [16]).

Oprozomib (ONX 0912) is a new orally bioavailable drug structurally analogue of carfilzomib. Like the latter, oprozomib is a potent, selective, irreversible proteasome inhibitor. In patients with advanced refractory solid tumors, MTD is 150 mg daily [38].

Several other proteasome inhibitors are currently in phase I or in a preclinical stage (Table 1, Figure 1). Future studies will be able to identify those that could represent real promises in the treatment of MM.

## 4. NOVEL IMiDs

Thalidomide is a racemic glutamic acid analogue, consisting of S− and R+ enantiomers that interconvert spontaneously under physiological conditions thanks to hydrolysis occurring in the liver. The S− form potently inhibits release of tumor necrosis factor (TNF alpha, which regulates apoptosis) from peripheral mononuclear blood cells and is responsible of immunological effects [39].

Thalidomide and its analogs lenalidomide and pomalidomide are effective agents in MM, known for their antiangiogenic and immunomodulatory properties thus to target at the same time neoplastic plasma cells and MM microenvironment. Recently, their molecular target has been identified in the protein complex of cereblon ubiquitin ligase that binds transcription factors of Ikaros family (IKZF-1, IKZF-3) [40–42].

Although lenalidomide arises from thalidomide, the mechanism of action can be different since thalidomide can revert lenalidomide resistance due to aberrancies in Wnt/*β*-catenin pathway. Under this perspective, in a phase I/II trial the combination of thalidomide and lenalidomide with dexamethasone was investigated in relapsed/refractory MM patients. After the phase I established the MTD in 25 mg lenalidomide/100 mg thalidomide/40 mg dexamethasone, 64 patients with a median of four prior lines of therapy were enrolled in the phase II of the study. Preliminary results were presented at 2013 ASH meeting by Dr. Shah.

Another strategy to increase the therapeutic effect of lenalidomide is based on its immunological properties, since lenalidomide seems to expand and activate natural killer (NK) cells. On the other hand, myeloma cells upregulate class I antigen of the major histocompatibility complex (MHC) that are ligands for inhibitory killer immunoglobulin-like receptors (KIR) and in this way are able to avoid NK cell killing [43]. IPH2101 is a monoclonal antibody against common inhibitory KIR that increases NK cell activity through inhibition of KIR-ligand interaction. After a phase I showing that IPH2101 is safe [44], preliminary results indicated that this drug can be safely and efficiently combined with lenalidomide, as suggested by Dr. Cohen at 2013 ASH meeting.

However, the most exciting new IMiD in the setting of relapsed/refractory patients is certainly pomalidomide. After the pioneer studies of Mayo Clinic [45], Richardson et al. have recently published a study dedicated to find the MTD, safety, and efficacy of pomalidomide, in 38 patients previously exposed to both bortezomib and lenalidomide (double refractory). With an MTD of 4 mg per day on days 1 to 21 of each 28-day cycle, with or without dexamethasone (40 mg/week), ORR was 42%, including 21% ≥ PR or better and 3% CR. Toxicity was predictable and manageable, with less than 5% of peripheral neuropathy and venous thromboembolism [17].

Preliminary data of the phase 2 study MM-002 have been presented by Dr. Jagannath at 2013 ASH meeting: 221 double-refractory MM patients were randomly assigned to receive either pomalidomide alone at 4 mg days 1–21 or in combination with low-dose dexamethasone (40 mg/week, 20 mg for patients over 75 years, pom + loDex). The ORR in the pom + loDex arm was 43%, with 37% achieving at least a PR. Median PFS and OS were 4.6 months and 16.5 months, respectively, in the pom + loDex arm versus 2.6 mos and 13.6 mos in the pom alone arm. Adverse events led to at least one dose reduction in 26% of patients, with neutropenia being the most common grade 3/4 adverse event. In the pom + loDex arm, grade 1-2 neuropathy occurred in 7% of patients.

Recently, San Miguel et al. reported the final results of a phase 3 trial (MM-003) comparing pomalidomide plus low-dose dexamethasone versus high-dose dexamethasone alone (hiDex) in 455 patients with relapsed or refractory multiple myeloma previously treated with lenalidomide and bortezomib (Table 3). In double refractory patients, PFS was 3.2 versus 1.7 months for pom + loDex versus hiDex and OS was not reached versus 7.4 months, respectively. The rate of grade 3-4 anemia, thrombocytopenia, infections, and VTE was similar in the two arms but neutropenia was more frequent in the pom + loDex arm as well as febrile neutropenia [18].

A final analysis, presented at the 2013 ASH meeting by Dr. San Miguel, confirmed with a longer follow-up that pom + loDex is superior to hi-Dex in terms of response rate and survival, despite 56% of patients on hiDex arm crossed to the pom + loDex arm. In MM-003 45% of patients were aged >65 years: the combination of pom + loDex significantly improved ORR also in elderly patients. Duration of response was significantly longer for pom + loDex versus hiDex in patients aged >65 years and >70 years. The pom + loDex safety profile was consistent by age and study discontinuation due to adverse events was 6% for patients aged <65 years and 13% for patients aged >65 years.

The superiority in ORR and PFS of the pom + loDex combination over hiDex was maintained in patients with moderate renal impairment, with a baseline creatinine clearance <60 mL/min, accordingly to Dr. Dimoupolos' communication at the last ASH meeting 2013 (ASH 2013, abstract number 2939).

On the basis of previous reports indicating that the addition of clarithromycin to lenalidomide and dexamethasone could be associated with improved outcome [46], pomalidomide was tested in association with clarithromycin and low dose dexamethasone (ClaPD). Dr. Boyer presented preliminary data from 114 patients (relapsed or progressed after at least three prior therapies) at ASH 2013. ORR was 70% (with 6% CR and 17% VGPR). In most patients, PFS was sustained for >8 months that is more than double the PFS reported in patients treated with pom + loDex.

Pomalidomide was tested in combination with cyclophosphamide and prednisone in relapsed/refractory patients in 69 patients enrolled in a multicenter phase 1/2 trial. MTD was 2.5 mg/day pomalidomide, cyclophosphamide at 50 mg every other day, and prednisone at 50 mg every other day, for 6 28-day cycles, followed by pomalidomide-prednisone maintenance therapy. Thromboprophylaxis was recommended. In 55 patients treated at MTD, the ORR was 50% including 23% ≥ VGPR, median PFS was 10.4 months, and 1-year overall survival was 69%. At the MTD, grade 3 to 4 toxicities included anemia, thrombocytopenia, neutropenia with grade 3–5 infections, and thromboembolism. Treatment was discontinued for toxicity in 9% of patients [19].

However, the outcome after achieving novel agents' refractoriness is poor. At Mayo, 74 patients from among 183 patients who had relapsed after pomalidomide phase 2 trials were retrospectively evaluated. The most commonly used regimen was bortezomib based (36%), followed by autologous stem cell transplantation (13%), alkylator-steroid combination (11%), VDT-PACE (12%), and lenalidomide based (11%). The highest rate of objective response of PR or better (80%) was seen in patients treated with ASCT. Lenalidomide was active in a proportion of patients relapsing on pomalidomide, suggesting that a trial of lenalidomide in these patients could be justified [47]. In other cases patients should be addressed to clinical trials involving new drugs described below.

## 5. Drugs Interfering with Growth Pathways

### 5.1. AKT Inhibitors

After the demonstration that the PI3K/AKT pathway is constitutively active in MM, providing signals that induce proliferation, angiogenesis, and development of drug resistance, several preclinical studies have shown that PI3K/AKT inhibition was able to induce tumor inhibition and regression in cell-line and animal models [48].

Perifosine is an oral AKT inhibitor able to induce cytotoxicity of plasma cells even in the presence of bone marrow stromal cells (BMSC) that confer cell adhesion-mediated drug resistance (CAM-DR). In this perspective, perifosine has shown an in vitro synergism with bortezomib, dexamethasone, and doxorubicin and it has been evaluated in a phase I/II clinical study in combination with bortezomib and dexamethasone in 84 heavily pretreated MM patients, including patients refractory to bortezomib (73%) or to bortezomib and dexamethasone (51%). The selected dose of perifosine was 50 mg/day plus bortezomib 1.3 mg/m^2^ and low-dose dexamethasone (20 mg) was added if progression occurred on perifosine plus bortezomib alone (Table 3). In 73 evaluable patients, the ORR was 41% (65% in bortezomib-relapsed and 32% in bortezomib-refractory patients). Median PFS was 6.4 months, and median OS was 25 months. Therapy was generally well tolerated and toxicities, including gastrointestinal adverse effects and fatigue, were manageable with supportive care and dose reductions. Grade ≥ 3 toxicities included thrombocytopenia (23%), neutropenia (15%), anemia (14%), and pneumonia (12%) [20].

Perifosine has been also evaluated in a phase I trial in combination with lenalidomide in relapsed and relapsed/refractory MM. Thirty-two patients received escalating doses of perifosine 50–100 mg daily and lenalidomide 15–25 mg once daily on days 1–21 of each 28-day cycle, plus dexamethasone 20–40 mg weekly. MTD was not reached and the ORR was 73% (including 13% nCR and 10% VGPR). Median PFS was 10.8 months and median OS was 30.6 months. The most common grade 1-2 adverse events were fatigue, diarrhea and grade 3-4 neutropenia, hypophosphatemia, thrombocytopenia, and leucopenia. This study also suggests that the clinical efficacy of perifosine-lenalidomide-dexamethasone is positively associated with phospho-Akt since PFS was longer in patients with high immunostaining of phospho-Akt than those with low staining [21].

Afuresertib (previously GSK2110183) is a potent, orally available, ATP competitive inhibitor of all three isoforms of AKT. A phase I trial has shown that afuresertib is well tolerated with clinical activity as single agent in heavily pretreated MM patients. After the demonstration that adding afuresertib to bortezomib promotes cell death and inhibits phosphorylation of downstream proteins in preclinical models, a clinical trial had been designed to evaluate MTD, safety, tolerability, and response rate of afuresertib, bortezomib, and dexamethasone combination. Dr. Spencer presented promising preliminary data at last ASH 2013 that will be updated soon.

### 5.2. Histone Deacetylase (HDAC) Inhibitors

Since histone deacetylases (HDACs) regulate cell differentiation and survival, their inhibition results in caspase-dependent and caspase-independent apoptosis. In preclinical studies, HDAC inhibitors affected the adhesion-mediated drug resistance and inhibited tumor growth in xenograft animal models of human MM. Although HDAC inhibitors as single agents have modest activity in MM [49], their combination with other antimyeloma drugs is promising, in particular with bortezomib because proteasome inhibition cooperates with HDAC inhibition of aggresome formation, leading to significant impairment of protein turnover [50]. Moreover, bortezomib transcriptional activity is favored by chromatin remodeling, that represents the molecular basis of the increasing interest in combining bortezomib with inhibitors of histone deacetylase or histone acetyltransferases [30, 51].

Given this rationale, two global multicenter clinical trials (VANTAGE 088 and 095) assessed efficacy and safety of treatment with vorinostat plus bortezomib in patients with relapsed or refractory MM.

In the VANTAGE 088, 637 patients, with a median of two previous treatments, were randomized to receive bortezomib at 1.3 mg/m^2^, on days 1, 4, 8, and 11 together with oral vorinostat 400 mg (317 patients) or placebo (320 patients) given once daily on days 1–14 of each 21-day treatment cycle.

ORR was better in the vorinostat group than the placebo group (56.2% versus 40.6%, *P* < 0.0001) with 7.9% versus 5.3% of CR. Median PFS was 7.63 months in the vorinostat group and 6.83 months in the placebo group. Serious adverse events were equally distributed, and an equal percentage of patients discontinued treatment because of drug-related adverse events. However, by considering all grades, some side effects were more pronounced in the vorinostat group such as thrombocytopenia, diarrhea, nausea, and fatigue [22].

The synergistic activity of bortezomib with another pan-deacetylase inhibitor, panobinostat, was also investigated. In a phase Ib dose-escalation study, panobinostat was given orally thrice weekly every week in combination with bortezomib (21-day cycles) in 47 relapsed/refractory patients. After MTD was determined, additional 15 patients received treatment with a 1-week holiday of panobinostat, and dexamethasone was added in cycle 2. The MTD for panobinostat was 20 mg and ORR was 52.9% in the escalation phase and 73.3% in the subsequent phase. More grade 3 or 4 adverse events were in escalation phase than in the expansion phase, including thrombocytopenia, neutropenia, and asthenia [23].

This study provided the basis for a phase II clinical trial program called PANORAMA 2 (panobinostat or placebo with bortezomib and dexamethasone in patients with relapsed multiple myeloma) in patients who had a progression of disease on or within 60 days of the last bortezomib-containing regimen. In the first part of the study, patients received 8 three-week cycles of oral panobinostat (20 mg) 3 times per week on weeks 1 and 2, bortezomib in the classic schedule on weeks 1 and 2, and oral dexamethasone (20 mg) 4 times per week on weeks 1 and 2. Responsive patients were enrolled in the second part of the study, which consisted of 6-week cycles of panobinostat 3 times per week on weeks 1, 2, 4, and 5; bortezomib once a week on weeks 1, 2, 4, and 5; and dexamethasone the same day and the day after bortezomib until disease progression. Fifty-five patients were included in the study and 17 completed treatment phase 1 and entered treatment phase 2. The ORR was 34.5% in this population of bortezomib-refractory patients. One patient (1.8%) achieved a near-complete response, and 18 patients (32.7%) achieved a PR. Additional 18.2% achieved an MR with a total clinical benefit rate of 52.7%. Median duration of response was 6.0 months and median PFS was 5.4 months. OS was not reached after a median follow-up of 8.3 months. The most common grade 3/4 adverse was thrombocytopenia (63.6%), managed with dose reduction or platelet transfusions but none of the patients discontinued treatment because of thrombocytopenia. Other common AEs were diarrhea, fatigue, anemia, neutropenia, and pneumonia [24].

Based on this demonstration of synergism between panobinostat and bortezomib, a recent study has evaluated the safety and efficacy of the combination of panobinostat with carfilzomib in relapsed and refractory MM patients. Preliminary data from 44 patients were presented at ASH 2013. Eighty percent of them had received both an IMiD and a proteasome inhibitor and 14% were considered refractory to both. Four dose levels were evaluated. Average starting dose was 20/45 mg/m^2^ for carfilzomib and 30 mg for panobinostat. Maximum tolerated dose was not achieved with carfilzomib while panobinostat frequently required both dose reductions (62%) and discontinuations (21%). ORR was 64% (with 31% ≥ VGPR). Previous refractoriness to proteasome inhibitors and IMiDs did not affect ORR in total patient population. Median PFS was 6.8 months in the total population and 4.8 months in patients refractory to bortezomib. The most frequent grade 3 or 4 treatment-related adverse events were thrombocytopenia and neutropenia. Nonhematological side effects included grade 3 fatigue, diarrhea, dyspnea, and hypertension. There was also one death due to congestive heart failure with hemolytic-uremic syndrome (ASH 2013, abstract number 1937).

Although HDAC inhibitors are synergistic with proteasome inhibitors, the efficacy of the combination is lower than expected on the basis of preclinical studies. The reduced activity could be explained in part by the side effects due to a nonselective HDAC inhibition that is responsible for hyperacetylation of numerous protein networks in cells. Although the mechanism of synergism between HDAC inhibitors and bortezomib is not fully understood, the most important HDAC involved in the aggresomal formation is the HDAC6 and it should be considered the new target for inhibition. In addition, its selective inhibition could not only enhance potency, but also reduce the toxicities related to off-target effects of pan-HDAC inhibitors. One of the most promising selective HDAC inhibitors is ACY-1215, that is approximately 11-fold selective for HDAC6 over HDAC3.

Low doses of ACY-1215 combined with bortezomib induce apoptosis in MM cells and a significant delay of tumor growth and a significant prolongation of overall survival in 2 different xenograft SCID mouse models [52]. Based on these results, a study has been conceived in which ACY-1215 was tested alone (part 1, phase 1a) or in combination with bortezomib (part 2, phase 1b) in MM patients relapsed or refractory after at least two lines of treatment. In the phase 1a, 15 patients were treated at doses up to 360 mg orally on days 1–5, 8–12 schedule of 21-day cycle. No MTD was identified. Adverse events reported were elevated creatinine, fatigue, hypercalcemia, and upper respiratory infection (not attributed to ACY-1215). In the phase 1b, 22 patients received ACY-1215 on days 1–5, 8–12 with i.v. bortezomib on days 1, 4, 8, and 11 with dexamethasone per OS 20 mg on days 1, 2, 4, 5 8, 9, 11, and 12. Grade 3 or 4 gastrointestinal adverse effects were rare and hematologic adverse events were manageable with grade 3-4 thrombocytopenia observed in 19% of patients. In these heavily pretreated patients, the ORR rate was 25% with a clinical benefit rate (≥SD) of 60%. Preclinical and ongoing clinical trials are exploring the activity of ACY-1215 with carfilzomib and IMiDs, as anticipated by Dr. Vogl at the last ASH meeting.

A less mature trial is exploring the combination of escalating doses of ACY-1215 together with standard dose of lenalidomide and dexamethasone, accordingly to a communication at ASH 2013. Dr. Vorhees said that ACY-1215 was well tolerated at doses up to 160 mg on days 1–5, 8–12, and 15–19 and no DLT has been observed so far. ORR was 81%, including 1 CR and 3 VGPR. Most common adverse events, mainly grades 1 and 2, were fatigue (50%), upper respiratory tract infections (38.9%), and neutropenia (27.8%, ASH 2013, abstract number 3190).

### 5.3. Signal Transduction Inhibitors

A new identified target for treatment of cancer is the kinesin spindle protein (KSP), a microtubule motor protein critical to the function of proliferating cells. Filanesib (ARRAY-520-212) is a KSP inhibitor that induces aberrant mitotic arrest and rapid cell death. It has a preferentially activity on MCL-1 dependent cells including MM and it is not expected to be cross-resistant with other drugs. In a phase II study presented at last ASH by Dr. Shah, filanesib was tested either alone (at the dose of 1.5 mg/m^2^ for 2 days every 2 weeks) or in combination with dexamethasone (40 mg weekly). Thirty-two patients with six median previous treatments entered the phase I (filanesib alone) and 55 patients with eight median previous treatments were enrolled in the phase II (filanesib and dexamethasone). The ORR was only 16% and the duration of response was 8 months in the single agent arm and 5 months for the combination arm. Therefore, this study confirmed the lower response rate in respect to the expectations induced by the preclinical studies [53]. However, this study explored the importance of *α* 1-acid glycoprotein (AAG) plasma levels in predicting the response to filanesib. AAG is an acute-phase serum protein that increases during inflammation. It binds to ARRY-520 and is responsible for increased IC_50_ for ARRY-520 in vitro. By dividing patients according to their basal AAG plasma level, the study demonstrated that all responding patients belonged to the low-level group while none of the patients with high level of AAG responded to filanesib. AAG levels correlated also with duration of response and overall survival indicating that AAG is an important selection marker for filanesib. Phase 1 studies of combination of filanesib with bortezomib or carfilzomib or lenalidomide are ongoing and preliminary results are encouraging.

## 6. Monoclonal Antibodies

Forty-six antigens potentially targeted by antibodies have been described in MM. Therefore a long list of monoclonal antibodies (MoAbs) is being tested either in preclinical or in clinical studies. Three main mechanisms of action are recognized for MoAbs:

1. direct killing of the antibody;
2. antibody-dependent cellular cytotoxicity (ADCC), in which the binding of a MoAb to a specific target on tumor cells is responsible for a contact between tumor cells and effector cells;
3. complement dependent cytotoxicity (CDC), in which recruitment of C1q by IgG bound to the tumor cell surface triggers a proteolytic cascade to disrupt the target cell membrane.

The advantage of MoAbs treatment relies on their relative mild toxicity that allows their combination with chemotherapy or other biological agents to be used at lower doses thus reducing the toxicity of antimyeloma treatment.

### 6.1. Elotuzumab, Anti CS1

The cell surface glycoprotein CS1 is constantly expressed at high levels on CD138^+^ purified plasma cells obtained from MM patients and at low level in activated B, NK, CD8^+^ T cells, and mature dendritic cells but not in normal tissues or stem cells.

Elotuzumab is a humanized anti-CS1 MoAb that exerts its antimyeloma mainly through ADCC mediated by NK cells but no CDC [54].

A phase 1 trial, that explored escalated doses of elotuzumab in patients with advanced relapsed/refractory MM, showed that adverse events (cough, headache, back pain, fever, and chills) were generally mild to moderate in severity but the antimyeloma efficacy was modest (26.5% only had stable disease) [55]. However, several preclinical studies have demonstrated that elotuzumab inhibits MM cell adhesion to the stroma, thus reducing drug resistance [54], and that there is a synergism between elotuzumab and other antimyeloma drugs, in particular bortezomib and lenalidomide [56]. Based on this, elotuzumab was administered together with bortezomib in a phase I/II trial in relapsed/refractory MM. An objective response was observed in 48% of evaluable patients with a median time to progression of 9.4 month. The most frequent grades 3 to 4 adverse events were lymphopenia and fatigue. Two elotuzumab-related serious adverse events (chest pain and gastroenteritis) occurred in one patient [57].

The combination of elotuzumab with lenalidomide seems to be more effective. A phase I/II study of combination of elotuzumab, lenalidomide, and dexamethasone has shown encouraging response rates in relapse/refractory MM setting [58], as recently updated at 2013 ASCO meeting [59]. In the phase I of this study, 25 patients were treated with elotuzumab 5, 10, or 20 mg/kg in 28-day cycles using standard 3 + 3 dose-escalation design. In the phase II study, 73 patients were treated with lenalidomide 25 mg/day on days 1–21 and dexamethasone 40 mg/weekly and, according to the number of previous treatment, were stratified to receive elotuzumab at the dose of 10 or 20 mg/kg i.v. on days 1,8, 15, and 22 in cycles 1 and 2 and on days 1 and 15 in cycles ≥ 3 (28 day cycles). In the phase II cohort the ORR was 84%, with higher rate observed with elotuzumab 10 mg/kg versus 20 mg/kg (92% versus 76%). CR/stringent CR was recorded in 14% of patients receiving elotuzumab 10 mg/kg versus 11% in those receiving 20 mg/kg. VGPR was obtained in 50% of patients treated with the lower dose versus 38% of patients treated with higher dose of elotuzumab. In addition, median PFS was longer in elotuzumab 10 mg/kg arm: 33.0 months versus 18.6 months. Elotuzumab was well tolerated in combination with lenalidomide/dexamethasone such that 52% of patients received therapy for ≥18 months. Most common grade 3/4 adverse events included anemia, thrombocytopenia, lymphopenia, and neutropenia without significant differences between the two arms and occurred during first 18 months of therapy.

These findings prompted 2 phase III trials of elotuzumab 10 mg/kg with lenalidomide/dexamethasone, which are currently ongoing for both relapsed/refractory MM (ELOQUENT-2) and previously untreated MM patients (ELOQUENT-1).

### 6.2. Daratumumab, Anti-CD38

Daratumumab is a human CD38 MoAb with broad-spectrum killing activity. Daratumumab has multiple mechanisms of action, including apoptosis and modulation of CD38 enzymatic activity, CDC, ADCC, and antibody-dependent cellular phagocytosis. In preclinical studies, daratumumab was able to kill myeloma cells and to enhance the activity of other MM treatments. Ongoing clinical trials are investigating the safety of daratumumab in combination with bortezomib or lenalidomide and dexamethasone in patients with relapsed or refractory MM.

In a study presented at ASH 2012 but not yet published, the drug was tested in 32 patients relapsed or refractory to at least two previous regimens. Doses ranging from 0.005 to 24 mg/kg were given weekly for 8 weeks for the first 16 patients and then biweekly for 16 weeks. In 26% of patients, an infusion-related reaction was observed during first full-dose infusion but without apparent relationship between dose and infusion-related reactions. Six patients across different doses experienced grade 3-4 adverse events that were related to treatment such as anemia, thrombocytopenia, bronchospasm, transaminases increase, and cytokine release syndrome. However, MTD was not reached. Daratumumab showed dose-dependent efficacy. Eight of 12 patients receiving ≥4 mg/kg daratumumab had at least a minimal response (ASH 2012, abstract number 73).

Dr. Arkenau presented at last ASH preliminary data on 11 patients indicating that the combination of daratumumab in a dose escalation design, given twice a month together with standard dose lenalidomide and dexamethasone, is safe and effective in relapsed or refractory MM patients. In this study, daratumumab at doses up to 16 mg/kg has been well tolerated (the MTD has not yet been reached) and in combination with lenalidomide and dexamethasone induced in all patients a reduction of M-component that was significant in 8 up to 11 patients (3 CR, 2 VGPR, 3 PR). The most frequent adverse events were neutropenia, gastrointestinal symptoms, bone pain, and muscle spasms. The daratumumab pharmacokinetics profile was not affected by lenalidomide and dexamethasone.

### 6.3. Tabalumab, Anti-BAFF

Tabalumab is a MoAb directed against membrane-bound and soluble B-cell activating factor (BAFF), a survival factor for MM. Preclinical studies have indicated an antimyeloma activity together with inhibition of osteoclastogenesis. Preliminary data have been presented at ASH 2012 and not yet published. In a phase I study, 20 relapsed/refractory MM patients were treated with a dose escalation of tabalumab (1, 10, 30, 100, or 300 mg on day 1, cycles 1–3, 5, and 7) together with bortezomib at standard biweekly dose (1.3 mg/m^2^). In the expansion phase, 28 patients received tabalumab at 100 mg. Grade 3/4 toxicities included peripheral neuropathy, pneumonia, diarrhea, gastrointestinal hemorrhage, musculoskeletal pain, thrombocytopenia, neutropenia, and anemia. The ORR was 45% including 2 CR. Response was associated with lower baseline serum BAFF or IL-6 levels. Median duration of response was 7.3 months and median TTP was 4.9 months (ASH 2012, abstract number 447). A multicentric randomized phase 2/3 clinical trial is ongoing to evaluate efficacy and tolerability of dosage 100 versus 300 mg on day 1 associated with bortezomib 1.3 mg/m^2^ on days 1, 4, 8, and 11 and dexamethasone 40 mg on days 1, 2, 4, 5, 8, 9, 11, and 12.

### 6.4. Indatuximab, Anti-CD138

Indatuximab ravtansine is an antibody-drug conjugate designed to deliver the maytansinoid cytotoxic agent, DM4, specifically to CD138+ expressing tumour cells. Indeed, CD138 is highly expressed in MM and more specific to identify neoplastic plasma cells than CD38. After binding to CD138, indatuximab ravtansine is internalized and processed in the lysosome to release lipophilic DM4 metabolites that inhibit tubulin polymerization and thus to induce cell cycle arrest and apoptosis.

Preliminary clinical data have been presented at the last ASH meeting. After a phase I dose escalation study (80, 100, and 120 mg/m²) to determine DLT and MTD, the drug has been evaluated in a phase II in a cohort of 37 patients. Indatuximab ravtansine was administered in a 28-day cycle on days 1, 8, and 15 together with lenalidomide (25 mg/day for 21 days) and low dexamethasone (40 mg/day, days 1, 8, 15, and 22). MTD was defined as 100 mg/m² with anemia and mucositis reported as dose-limiting toxicities. Among the 15 evaluated patients, the ORR was 73%, including 2 CR and 4 VGPR. This ORR was maintained in lenalidomide refractory patients and was even higher (89%) in 8/9 patients treated at MTD (ASH 2013, abstract number 758).

## 7. Conclusions

Many new encouraging studies support an optimistic view of the future, although many dark zones remain in the pathway to cure MM. The landscape of treatment of MM is changing thanks to the new developments in understanding the biology of disease and the utilization of the new drugs although sometimes it is hard to figure out exactly the results of the most published studies in the salvage setting since enrolled cohorts are often heterogeneous and include patients with different prognosis. The new therapeutic scenario is dominated by novel therapies where drugs are able to target specific mechanisms of neoplastic cell growth. However, myeloma is quite a heterogeneous disease and neoplastic plasma cells can use several metabolic pathways in order to take a growth advantage. In addition, several studies have shown that different neoplastic clones may emerge in different phases of disease and it is possible that each clone has a different profile of drug sensitivity. It is therefore possible that each of the drugs is effective only in a subgroup of patients and within this group only during a specific phase of disease. To find the specific field of activity of each new drug will be a challenge for future studies.

Another critical point is the awareness that the new drugs often act with mechanism of action that are different from chemotherapeutic drugs, but clinicians are still accustomed to use them as chemotherapy. In many studies the object is to find the MTD while it is becoming clear that for many biological agents not always “the more is better.” One example is elotuzumab: a lower dose yielded better results. New methods for measuring biological drug efficacy should be developed in the future.

In the era of the new drugs, however, it should be underlined that the “old” chemotherapy drugs still maintain a significant efficacy against myeloma and that in many cases chemotherapeutic drugs represent the backbone to which the new drugs should be added. A fine tuning of this combination is another skill that clinicians should acquire in the future.

Dealing with relapsing patients, quality of life should be one of the most important goals to be achieved. However, this kind of evaluation is often lacking in many studies and the tools for measuring it are not widely known.

In other cancers, the identification and validation of biomarkers have leaded to improvement of outcome while in MM a precise definition of high risk is still lacking. Novel biomarkers predictive of outcome include cereblon and Ikaros for IMiDs and AAG for Akt inhibitors. However, a better definition of the prognostic profile of the patients could help in interpreting the trials and could be useful to clinicians to identify treatment more appropriate for high-risk patients.

## Figures and Tables

**Table 1:** Regimens containing bendamustine and novel agents.

Combination with novel agents: bortezomib					ORR	PFS	Reference
phase I-II	Bendamustine	90 mg	i.v.	Day 1 and day 4 every 28 days for up 8 cycles	52%	not reached	Berenson et al., 2013 [7]
	Bortezomib	1.0 mg/m^2^		days 1, 4, 8, and 11 every 28 days for up 8 cycles			

BVD phase II	Bendamustine	70 mg	i.v.	Day 1 and day 4 every 28 days for up 8 cycles	76%	9.7 months	Ludwig et al., 2014 [8]
	Bortezomib	1.3 mg/m^2^	i.v.	Days 1, 4, 8, and 11 every 28 days for up 8 cycles			
	Dexamethasone	20 mg	i.v.	Days 1, 4, 8, and 11 every 28 days for up 8 cycles			

BVD phase II	Bendamustine	70 mg	i.v.	Day 1 and day 8 every 28 days for up 6 cycles	71.5%	16.5 months	Offidani et al., 2013 [9]
	Bortezomib	1.3 mg/m^2^	i.v.	Days 1, 4, 8, and11 every 28 days for 2 cycles and then days 1, 8, 15, and 22 for 4 cycles			
	Dexamethasone	20 mg	i.v./OS	Days 1-2, 4-5, 8-9, and 11-12 every 28 days for 2 cycles and then on days 1, 8, 15, and 22 for 4 cycles			

BPV phase II	Bendamustine	60 mg	i.v.	Day 1 and day 2 every 28 days for up 7 cycles	69%	11 months	Ponish, 2012
	Bortezomib	1.3 mg/m^2^	i.v.	Days 1, 4, 8, and 11 every 28 days for up 7 cycles			
	Prednisone	100 mg	i.v.	Days 1, 2, 4, 8, and 11 every 28 days for up 7 cycles			

Combination with novel agents: IMiDs							

BTP phase I	Bendamustine	60 mg	i.v.	Day 1 and day 2 every 28 days up to maximum response or MTD	80%	11 months	Pönisch et al., 2008 [10]
	Thalidomide	50 or 100 or 200 mg	per OS	Days 1–21 every 28 days up to maximum response or MTD			
	Prednisone	100 mg	i.v./per OS	Days 1, 2, 4, 8, and 11 every 28 days up to maximum response or MTD			

BTD phase I-II	Bendamustine	60 mg	i.v.	Day 1 and day 8 every 28 days up to progression	46%	19 months	Yong et al., 2013 [11]
	Thalidomide	100 mg	per OS	Days 1–21 every 28 days up to progression			
	Dexamethasone	20 mg	i.v./per OS	days 1, 8, 15, and 22 every 28 days up to progression			

BLD	Bendamustine	75 mg	i.v.	Day 1 and day 2 every 28 days up to progression	76%	6.1 months	Lentzsch et al., 2012 [12]
	Lenalidomide	10 mg	per OS	Days 1–21 every 28 days up to progression			
	Dexamethasone	40 mg	i.v./per OS	Days 1, 8, 15, and 22 every 28 days up to progression			

BLP	Bendamustine	75 mg	i.v.	Day 1 and day 2 every 28 days up to progression	76%	48% at 18 months	Ponish, 2013
	Lenalidomide	25 mg	per OS	Days 1–21 every 28 days up to progression			
	Prednisone	100 mg	per OS	Days 1–4 every 28 days up to progression			

**Table 2:** The most active proteasome inhibitors currently tested in relapsed/refractory myeloma patients.

Drug	Bond to proteasome	Route of administration
Carfilzomib	Reversible	i.v.
Marizomib (NPI-0052)	Irreversible	i.v.; oral
Ixazomib (MLN9708/MLN2238)	Reversible	i.v.; oral
Oprozomib (ONX0912)	Irreversible	oral
Delanzomib (CEP-18770)	Reversible	i.v.; oral

**Table 3:** New generation drugs in monotherapy or combined to novel agents.

Drug	Association to novel agents	Phase	Dosage	*N*	ORR	Reference
Carfilzomib	Monotherapy	2	20–27 mg/m^2^	257	36	[13]
Carfilzomib	In combination with lenalidomide and dexamethasone	1/2	27 mg/m^2^	51	78	[14]
Carfilzomib	In combination with pomalidomide	1/2	27 mg/m^2^	82	70	[15]
Ixazomib	Monotherapy	1/2	2.97 mg/m^2^	32	26	[16]
Pomalidomide	Monotherapy	1/2	4 mg	38	42	[17]
Pomalidomide	In combination with high dose dexamethasone	3	4 mg	455	31	[18]
Pomalidomide	In combination with cyclophosphamide and prednisone	1/2	2.5 mg	69	50	[19]
Perifosine	In combination with bortezomib	1/2	50 mg	84	41	[20]
Perifosine	In combination with lenalidomide	1	50–100 mg	32	73	[21]
Vorinostat	In combination with bortezomib	3	400 mg	637	56.2	[22]
Panobinostat	In combination with bortezomib	1b	20 mg	47	52.9	[23]
Panobinostat	In combination with bortezomib and dexamethasone	2	20 mg	55	34.5	[24]
